# An enjoyable involvement: A qualitative study of short-term study abroad for nursing students

**DOI:** 10.1371/journal.pone.0249629

**Published:** 2021-04-02

**Authors:** PingRu Hsiao, ChunChih Lin, ChinYen Han, LiHsiang Wang, LiChin Chen

**Affiliations:** 1 Department of Nursing, Chang Gung University of Science and Technology, Taoyuan, Taiwan; 2 Chang Gung Memorial Hospital, Taoyuan, Taiwan; 3 New Taipei Municipal TuCheng Hospital, Taipei, Taiwan; University of Macau, MACAO

## Abstract

This study aimed to explore the experiences of nursing students who have undertaken a short course in an overseas educational system to generate theoretical understanding of the experience of studying abroad. Learning in the health professions needs to considered in the context of globalization as a way of sharing knowledge and taking responsibility as world citizens. Studying abroad provides students with an opportunity to develop global health care competencies. A symbolic interactionist approach was adopted to gain insight into how the students constructed the meaning of studying abroad. The study was conducted at a university with 2 campuses in Taiwan. A purposive sampling strategy was employed to recruit 14 participants. Data were collected from August to November 2017 via individual in-depth interviews. A semi-structured interview guide was used. Qualitative content analysis was employed to analyse the data. Each interview was audiotaped and transcribed verbatim. Most students identified both positive and negative experiences related to their professional and personal development and needs. They demonstrated motivation to leave their comfort zone and enter a challenging environment as well as a high level of adaptability. The experience enabled them to see themselves as part of the world and expanded their learning in positive ways. On completion of their course, they encouraged others to experience this enjoyable once-in-a-lifetime opportunity.

## Introduction

Learning in the health professions needs to be considered in the context of globalization as a way of sharing knowledge and taking responsibility as world citizens [1]. Studying abroad provides students with an opportunity to develop global health care competencies [2] and is encouraged as part of nursing education to enable students to experience nursing culture in different health care and/or educational systems. It has both professional and personal benefits for students and enhances their understanding of the international health industry [3,4]. Studying abroad also has a positive influence on the development of cultural competence in nursing care [5], enhances personal growth as the result of increased independence and confidence [6] and contributes to professional development [7]. The impact of international experience on the development of knowledge and practice is enhanced by students’ expectations, engagement, and critical reflection [8]. It also contributes to their transition to independence and has a long-term positive effect on their development as care professionals [9].

A study by Brown et al. [10] identified the three main positive factors influencing a student’s decision to study abroad as the desire to travel, to experience other cultures and to work in different health care settings. Study abroad may also be influenced by a student’s religious beliefs [11]. Factors deterring students from studying abroad include health and safety concerns, cultural shock [12], cost and language barriers [13], separation from family and friends [1] and worry about family responsibilities [14].

Most study abroad programs are organized as a summer course, which allows more time to make travel arrangements. Although longer programs have a greater impact on a student’s education [15], they do not necessarily lead to a significant increase in competence [16]. A short period of study abroad in the form of a micro-semester in nursing curricula was reported to help students expand their views on global health care issues [2]. An evaluation of a four- or eight-week study abroad experience was found to have a significant impact on students’ cross-cultural adaptation [17]. Although this is a short amount of time to explore another culture, it is sufficient for students to develop multicultural knowledge and awareness [18] and cultural competence in care [2]. Studying abroad can be meaningful regardless of the length of the experience. The most important factor is that the program is carefully designed to facilitate student’s experience. Chen [13] identified language and culture as vital considerations in the organization of a study abroad program. Thorough preparation, a small study group, and a longer timeframe can improve students’ learning [19].

The Taiwan Nursing Accreditation Council (TNAC) has identified nursing eight competencies that should underpin the curricula of all nursing programs in Taiwan. These competencies in nursing are: (a) critical thinking and reasoning, (b) general clinical skills, (c) basic biomedical science, (d) communication and team work capability, (e) caring, (f) ethics, (g) accountability and (h) lifelong learning. These competencies guide and ensure that nursing education achieves expected learning outcomes in different levels and produces “a nurse who is trusted by patients and their families” [20]. These competencies encapsulate key ability necessary to enter the nursing field in the future and they are characterised as soft power in nursing. In addition to the nursing professional knowledge and skills of nursing competencies, the nursing soft abilities as power, such as critical thinking, communication skill and life- long learning, influence and promote nursing students engaging in the nursing field continually. These soft abilities as an alternative to understand the complex interactions of client’s health and care situations. Although multicultural competence is not included in these competencies of TNAC, there can be little doubt of its importance in the context of globalization. Study abroad has been shown to have a significant impact on nursing students’ professional and personal development [4].

In this study, students adapted their behavior in response to the overseas environment. The focus of inquiry was on how students defined the situation of being in a foreign country, how they perceived the study abroad experience, and how they used a shared understanding of studying abroad in constructing its meaning. The study aimed to explore the experiences of nursing students participating in a short-term course in an overseas educational system and generate theoretical understanding of the experience of studying abroad.

## Materials and methods

### Study design

Symbolic interactionism (SI) [21] is concerned with meaning, language, and thought. It focuses on interaction and the meanings of events to participants’ definitions of situations. Thus, SI emphasizes the processes through which people define, act on, and use language in their interactions with the environment. SI provides a theoretical framework for studying how individuals interpret objects and events that they encounter in their lives and how a process of interpretation leads to action in a specific situation. It provides a perspective on and a means of understanding how those in a particular situation define reality.

In this framework, students’ behavior while overseas is seen to result from their adaptation to the environment (overseas). The focus of inquiry was on how the self (nursing students) defined a situation (being in a foreign country) and reproduced social actions (students’ views of studying abroad), and how the self (nursing students) shared an understanding of studying abroad in constructing its meaning. SI focuses on interaction (students dealing with the experience of study abroad) and on the meanings of events. It emphasizes the processes involved in defining, acting on, and using symbols (language) through interactions and responses (thought) to interactions with the environment (study in foreign country).

#### Setting and participants

The study was conducted at a university with 2 campuses in Taiwan. Approximately 800 nursing students graduate each year, of whom 64 students are selected to participate in study abroad programs. Students choose a study abroad program depending on the purpose of the program and their personal interests. Purposive and convenience sampling strategies were used to recruit 14 participants. The inclusion criteria were: (1) currently registered as a student at the university, (2) selected for a 4-week study abroad program, (3) chose to study at an Australian university, and (4) consented to having their responses audiotaped. The exclusion criteria were: (1) having withdrawn from a formal course and (2) being selected for a 2-week program of studying abroad. Ten participants, including undergraduate and post-graduate students, consented to be interviewed before and after their overseas trip, and 4 participants were interviewed after their overseas learning experience (S2 Table).

Students who are interested in study abroad programs are required to undergo a screening process that includes a written review and face-to face interview in order to be accepted into a training session. The training session emphasizes language, customs, culture, and the daily lifestyle of the target county. Potential participants in this study were approached after the training session from among the successful applicants. Students selected for study abroad receive substantial finance support from the school and 2 academic credits for their elective course in the nursing curriculum.

### Ethical considerations

Organizational permission was first obtained from the university that served as the study site. Ethical approval for the study was obtained from the Institutional Review Board of Chang Gung Medical Foundation (Approval no. 201601967B0). Potential participants were invited verbally by the PI. When a potential participant demonstrated an interest in participation, she/he received an information sheet and a consent form. Prospective participants were given time to read the information and think about the study before making a final decision to participate. The participants were fully informed about the study, including the purpose, procedures for data collection, potential risks and benefits, time commitment, and how their rights to privacy and anonymity were protected. They were advised that participation was entirely voluntary and that they had the right to withdraw from the study at any time without penalty. The decision to participate or not would in no way impact their participation in the exchange program. No coercive or deceptive tactics were used to encourage participation. Participants’ written consent was obtained before the interviews were conducted. The data, in the form of interview transcripts, were stored in the PI’s password-protected computer. The data were deleted within 3 years of the study’s completion according to IRB requirements. In this report, participants’ anonymity has been preserved by assigning each student (S) a number (e.g., S1, S2, etc.).

#### Data collection

Data were collected from August to November 2017 via individual in-depth interviews conducted by the principal researcher (PI). A semi-structured interview guide (S1 File–Interview guide) was used to ensure both consistency and flexibility in the data collection and to maintain focus and avoid any bias toward the researcher’s specific areas of interest. Ten interviews were conducted before and after the participant’s overseas trip to gain insight into their expectations and actual overseas experiences. Four participants were interviewed after the trip because time restricted after admission list released. Interviewing in the participants’ preferred language of Mandarin maximized the data quality. Shared culture and language between the interviewer/researcher and the participants facilitated in-depth understanding and interpretation of verbal and non-verbal cues. The interviews commenced with an open question, “Could you please share your experience of overseas study?”. Further questions varied depending on the participants’ responses. Each interview lasted 45–60 minutes. The interviews were audiotaped to secure an accurate account of the conversations. Research team members, including the PI, were not involved in the process by which students were selected for study abroad programs. Potential participants were approached only after their applications had been accepted.

### Data analysis

Qualitative content analysis [22] was used to analyze the data. Each interview was transcribed verbatim. Data organization started with the management of coded files. Each file contained interview transcripts and audiotapes. All of the interview transcripts were merged into one file for general reading purposes. The audio recordings were listened to repeatedly to gain a deeper insight into the data.

The analysis was conducted in four stages, including manifest and latent analysis. In the de-contextualization stage, all of the data were carefully read line by line and preliminary coding was undertaken. The meaning of each code was defined through frequent discussions among the members of the research team. In the re-contextualization stage, all codes with similar meanings were grouped and assigned to a subcategory. The group and its name were considered in the initial texts to ensure the meaning of the text. In the next phase, all homogeneous categories were identified from the list of code definitions from the contextualization stage. The final stage, compilation, involved the development of a realistic conclusion with which all team members were in agreement.

### Trustworthiness

Four criteria guided the quality assessment: credibility, transferability, dependability, and conformability [23]. The trustworthiness of the study process was ensured by the following factors: (1) the researchers were experienced in the field; (2) as many participants as possible were selected; (3) the participants were encouraged to share their perspectives and experiences; (4) all research team members agreed on the themes and read the report; (5) the findings were presented using direct quotes; (6) the descriptions contained rich data; (7) the data were obtained by a single and highly trained investigator; (8) the interviews were audiotaped and transcribed verbatim to ensure accuracy; (9) an experienced researcher identified major categories, ensuring the credibility of study results and processes; (10) an interview guide was used to guarantee consistency; and (11) fieldnotes recorded the study process. The study process was therefore considered rigorous.

## Results

The participants were 14 female undergraduate and post-graduate students aged 21–37 years (average 24 years). Participants’ demographic information is presented in in S2 Table. Three themes emerged from analysis of the data: the self in relation to the world, diversifying learning and a once-in- a- lifetime experience. The overall conclusion was that participants enjoyed the study abroad experience (see S1 Table). In the following presentation of results, participants’ responses have been anonymized, as discussed earlier.

### The self in relation to the world

The self is the center of one’s world. Placing oneself in a different environment can provide an opportunity to gain a deeper understanding of oneself and the world [21].

#### Relating to the world

An individual’s worldview is constructed from his or her past experiences and shapes his or her values and interactions with the environment. Because people are social creatures, individuals seek to connect to the environment with a purpose, and the decisions they make predict the eventual achievement of the object [21]. When the environment is unfamiliar, individuals must be willing to modify or change their values and actions in their interactions with others in that environment.

The study participants realized that the world (environment) they were entering would influence their behavior and they would need to make changes to ensure their behavior was acceptable in this new environment as they interacted with others. These changes represented the line between knowing and doing (conducting), as S1 noted in the following statement. The environment was not going to change to meet their needs; rather, they had to adapt to the environment. As they did so, they became immersed in it.

… I felt a bit nervous and embarrassed at the beginning of class because I was surrounded by foreign faces. You were in the minority and knew no one in class. I needed great courage to raise my hand or say something during classes which was different to my study attitude. Nothing would be changed for you unless we changed first. After the second week, I started to preview the class content before attendance, which I had never done before … (S1).

#### Adapting to a new environment

After the participants entered the new environment, they attempted to make the best of it. Although there were no standards or rules for them to follow, their understanding of the unfamiliar environment grew out of their close relationships with others. Understanding what others say is crucial to effective communication, as is the ability to voice one’s views and opinions. In other words, language is a vital tool for interacting with the environment, learning from others, communicating with others, and expressing oneself. The study participants were aware that, because English was their second language, they needed to adopt a positive attitude towards overcoming their limitations in order to engage in learning situations. They knew they could not wait for people to actively approach them, and that their language limitation was a weakness that needed to be improved. Being actively involved in the environment requires a positive approach to dealing with situations. On the other hand, people may withdraw from a situation or an environment into their own inner world, thus reducing their contact with that world. Clearly, the latter response restricts the potential outcomes of learning abroad. In this study, participants were well aware of their weaknesses and strengths and modified their actions to adjust to the environment.

… You had to know what people said. It made me listen to people more seriously. You ignore problems at the beginning of studying abroad and solve them autonomously. The more you practiced and experienced, the more you felt confident and not shy…You try to hide from the discussion in the classes. There has no way to dodge changing situations…(S2)

#### Gaining a deeper understanding of oneself

The process of interacting with their inner worlds was initiated by a stimulus. The interaction consisted of comparing and judging stimuli from past and present experiences. The outcomes of comparison and judgment were not right or wrong, positive or negative, or better or worse. The participants in this study gained a deeper understanding of themselves, situations, and the world through the process of comparison and judgement. An itemized teaching-learning strategy between the hosts and the study abroad environments enabled the participants to reach a greater understanding of themselves. Knowing and experiencing things from others informed the participants’ own knowledge of their weaknesses and strengths.

Participants understood situations better through the process of comparison and judgment. They soon realized that they would not succeed without changing. They modified their perceptions and actions to interact with the environment, which is known as adjustment. They identified that widening their worldview was a part of growing up.

…We tolerated things and respected people who were more mature than us. It could be said that we rarely had our own thinking or ideas appreciated. We were obedient, shy, and polite in many peoples’ first impression. . . . Things became difficult for us compared to living in Taiwan. Therefore, you were forced to grow up. Our teaching-learning was like a standardized answer was expected, which was different. We had to be more visible and adaptable in a Western country…(S3).

#### Acquiring a new vision

By immersing themselves in their new environment, participants acquired a new vision of things. When they confronted something that was completely different, they engaged in a process of comparison and judgment. The new experiences challenged their previous thinking. Through comparison with their previous experiences, they identified their new way of thinking as an international vision. Although they found this vision hard to define, they recognized that it influenced how they thought about the next step in their personal and professional growth.

I used to think my view on things was international. Actually, it was not what I thought after the trip abroad. I did not know how to specifically express my international vision in words …The journey widened my worldview. Meeting people, seeing and experiencing things, I realized that the world was so much fun. My future career and personal life would not be only in a hospital, I had various options… (S4).

### Learning diversity

Motivation for change is essential to achieve learning diversity. A wide range of experiences during the educational process builds self-confidence and passion for learning in the field.

#### Motivating oneself to change

For the participants, leaving a familiar environment meant leaving their comfort zone and motivated them to make a range of personal changes, which enhanced personal growth. They described the process of adaptation as one of *fitting in* to a new environment. A change in behavior was necessary to help them feel secure and avoid deviating from accepted social norms. Participants realized that overcoming their insecurity and interacting in the new environment was an opportunity for personal learning and growth.

The atmosphere of the environment compelled everybody to keep quiet and invisible like the Taiwanese student’s style of learning. When [I was] in an active learning environment, I would modify my learning attitude to be more proactive like everyone else. When the surroundings were passive, I would respond passively like everybody else. This was about fitting in (S5).

#### Inspiring self-confidence

When in their familiar environment, the participants reported, they had a more limited learning focus because they were not greatly challenged and took things for granted. In the new environment, learning became more diverse, with limited assistance from family or friends. Each issue had to be solved or managed independently, which can be seen as learning. The participants had to modify their attitudes and actions when interacting with and adapting to the new environment, and construct ways of dealing with new experiences. S6 described how she developed the confidence to acquire new information and a new vision from the things that happened. Participants felt that their perspectives had been broadened.

If you have had the chance to go abroad, you would see a different world. You would know that people are under the same sky but are living in different ways, which are varied and beyond our imagination. You would understand the importance of experiencing other lifestyles, and not be confined to your current life… (S6).

#### Reenergizing nursing education

The short timeframe of a study abroad program could be used to justify minimal gains in professional development. However, participants renewed their passion for nursing as a result of the discomforting experience of the host country’s working environment. The substantial new environmental stimuli, including people, incidents, objects, and situations, helped them to develop strengths. The participants opened their minds and managed their problems. This reflected positively on their attitudes toward challenges in an unfamiliar environment. Dealing with challenges reinvigorated the participants’ original intention to study nursing. The new environment improved their attitude toward challenges and motivated their interactions. The consequences were significant and reenergized their enthusiasm for nursing. Studying abroad was a remarkable, once-in-a-lifetime experience.

I was under pressure during the internship. Although I studied nursing voluntarily, learning was much like being forced to learn things.…After I returned home, I felt passionate about continuing my nursing education. I did not forget my original intentions for studying nursing… I feel that I have regenerated my enthusiasm for being a nursing student (S7).

### A once-in-a-lifetime experience

Although the reality of studying abroad in real life may have been vastly different from their expectations, imagination, or information obtained from others, it was a valuable once-in-a-lifetime experience.

#### Facing the reality

The participants experienced a direct barrier to learning due to their lack of fluency in English, which may have impacted their comprehension of the topic under discussion. This highlights one of the main difficulties associated with learning in an environment for those with English as a second language. Lack of fluency impeded their learning and affected their ability to express themselves accurately.

The style of having lectures and language had a great negative impact on my learning progression. My English, including vocabulary and phases, tended not to be used in daily life, so it failed to express the meaning of things. I needed more time to respond …(S8).

#### Confidence, personal growth, and knowing before doing

Becoming confident stemmed from personal growth following interaction with other people in a new environment. Participants’ satisfaction and confidence increased as their language skills improved. Interactions also provided learning opportunities and required them to adjust their attitudes towards connecting with the world. The participants became more confident as they received positive feedback from others. They knew what they had achieved and were motivated to set new goals, which taught them about planning. This was the first time in their lives that they had had to plan future goals, which was significant for their personal growth and maturity.

S9: I had learned more since I applied to this program. My attitudes towards things were more likely lazy and superficial. I started to modify my attitudes and that gave me a feeling of progression. I achieved my goal to study overseas and it was time to set the next goal and move forward.

#### Learning from the real world

Learning from the real world emphasizes the development of soft power. Soft power involves managing daily activity, although it is difficult to teach or learn in the classroom. Learning from the real world made up for the shortcomings of classroom learning; the world provided a practical scenario for personal growth. Soft power encapsulates respecting others, enhancing self-directed learning, being confident, and becoming an active learner and critical thinker. The real world was a grand lecture theater that cultivated the participants’ personal growth. They felt that maintaining distance from others was not a sign of rudeness but a way of showing respect. Maintaining distance enabled them to avoid stress while interacting with others. This feeling could only be experienced in a new environment and was better taught via firsthand experience.

S10: People were respectful to others. I thought they were civilized. People would keep their distance from others…I became confident; it may be because of the change in learning from passive to active. Once you developed the courage to say what you thought in class, you became confident.

## Discussion

The findings highlight the participants’ discovery of self and their achievements. Most of the students identified both positive and negative experiences related to their professional and personal development and needs. They demonstrated their motivation to leave their comfort zones and face a challenging environment. Most said they rapidly modified their learning attitudes and approaches to the new learning environment. They demonstrated considerable aptitude for adjustment. Studying abroad provided a chance for the students to discover *self*, to see themselves as a part of the world (*Relating to the world*) [24] and expanded their learning in positive ways (*Learning diversity*). The students became confident at critical thinking and became active learners. They also believed that different experiences could inform their own lives and taught them to understood the concept of learning by example (*Facing reality*). After they finished the course, they encouraged others to experience a once-in-a-lifetime opportunity as *an enjoyable involvement*. The experience of short-term study abroad was positive [25].

The literature indicates that students confront a range of challenges when they study in different educational contexts [26]. These include issues with language proficiency [27], cultural barriers [28], different learning styles, academic demands [29], perceived racism [30], homesickness, lack of assertiveness, and financial difficulties [31]. The students in this study faced similar challenges; however, the positive influences and learning outcomes were greater than the negative. The negative factors may have inspired the participants to cultivate personal growth as they left their comfort zones and had to adjust to new environments [32]. Furthermore, although they reported limited gains in professional development, they experienced personal growth by developing confidence, motivation to change, and *soft* power [33]. The study abroad experience fostered the development of *soft power* or ability to deal with the world [34]. Limited professional development was likely due to the short study period of four weeks. The students experienced a Western learning style that informed their expansion of personal growth and valued an interactive mode of learning [35]. Personal development has a significant impact on students’ motivation for life-long learning. This study’s findings indicated that geographic distance was a particularly difficult problem that affected the participants’ daily lives. It is challenging to change one’s lifestyle in a short period of time, and distance exacerbated the difficulty. It is easier to travel in Taiwan than in Australia, which have similar sized populations but are vastly different in area [36]. Distance is also highly related to time. More time is required to manage everyday activities such as grocery shopping. However, such inconveniences taught the students to plan ahead. At the same time, the maintenance of personal space demonstrated respect for others and helped to avoid stress in a crowd.

Although the students understood different learning styles, they did not advocate them. The shortness of their stay and the need for self-directed learning, such as preparing for lectures beforehand, might have contributed to their reluctance to advocate the Western learning style. However, students did appreciate and support Western teaching strategies. There are clear differences between Eastern and Western education styles. These differences reflect different attitudes towards social and individual education as the primary means of adapting to the environment. Cultural differences result in different approaches to social and individual learning [37]. The western learning style is characterized as independent, autonomous and hierarchical, whereas the eastern learning style is interdependent, harmonious and democratic. These differences result from social learning and individual learning as primary means of adapting to the environment. Therefore, it requires a long period of immersion in another culture to integrate the different characteristics of Eastern and Western learning styles, since they tend to be mutually exclusive. Overall, the students did not advocate the Western learning style, believing it limited their professional development when they only had a short study period.

## Conclusion

The students’ experience of achieving their goals can be characterized as an enjoyable involvement. They gained greater self-awareness and broadened their worldview. They transcended their preconceived ideas and expanded their educational boundaries. They engaged in introspection and developed self-confidence. They were reinvigorated and rediscovered their original reasons for pursuing careers in nursing. They also motivated themselves to pursue life-long learning. Participants described how their experiences would have a positive effect on their personal growth and professional lives in their future career as they shared their overseas learning experience with peers. Such an enjoyable involvement is clearly valuable and should be further developed as a means of enriching nursing students’ education. The results of this study also identified ways in which faculty can address potential issues in pre-travel orientation sessions and support learning activities during the experience.

### Study limitations

Only female nursing students were recruited at one university. The results may not reflect male students’ perspectives. Furthermore, this study was conducted at one university and study abroad nation, which raises concerns about the representation of the students’ perspectives on traveling in different countries or with different educational backgrounds.

## Supporting information

S1 Table An enjoyable involvement of studying abroad. (DOCX)

S2 Table Participants’ demographic characteristics. (DOCX)

S1 File Interview guide in English and Chinese. (DOCX)

## References

[1] Kent-Wilkinson A, Dietrich Leurer M, Luimes J, Ferguson L, Murray L. Studying abroad: Exploring factors influencing nursing students’ decisions to apply for clinical placements in international settings. Nurse Educ. Today. 2015; 35(8): 941–947. 10.1016/j.nedt.2015.03.012 25846198

[2] Garber JS, Saylor J, Johnson AN. Innovative Global Health Care Experiences for Nursing Students: Microsemesters and Internships. Nurs. Educ. Perspect. 2019; 40(5): 306–308. 10.1097/01.NEP.0000000000000559 31436695

[3] Smith K, Curry K. Is it worth it? Measuring the long term effects of an international experience for nursing student in Ecuador. J Community Health Nurs. 2011; 28 (1): 14–22. 10.1080/07370016.2011.539080 21279886

[4] Kelleher S. Perceived benefits of study abroad programs for nursing students: An integrative review. J Nurs Educ. 2013; 52 (12): 690–695. 10.3928/01484834-20131118-01 24256000

[5] Kokko R. Future nurses’ cultural competencies: What are their learning experiences during exchange and studies abroad? A systematic literature review. J. Nurs. Manag. 2011; 19 (5): 673–682. 10.1111/j.1365-2834.2011.01221.x 21749541

[6] Milne A, Cowie J. Promoting culturally competent care: the Erasmus exchange programme. Nurs Stand. 2013; 27 (30): 42–46. 10.7748/ns2013.03.27.30.42.e7215 23617062

[7] Knecht LD, Wilson KJ, Linton ME, Koonmen JM, Johns EF. Assessing student expectations and perceptions of a short-term international service-learning experience. Public Health Nurs. 2020; 7(1):121–129. 10.1111/phn.12669 31560808

[8] Aseno BA, Reimer-Kirkham S, and Astle B. In real time: Exploring nursing students’ learning during an international experience. IJNES. 2013; 10 (1): 227–236. 10.1515/ijnes-2012-0045. 24150212

[9] Heiberg IG, Dahl H, Ådnøy Eriksen K. How can studying abroad nurture nurse students’ intelligent kindness. Nurse Educ Pract. 2019; 41: 1–6. 10.1016/j.nepr.2019.102644 31704476

[10] Brown M, Boateng EA, Evans C. Should I stay or should I go? A systematic review of factors that influence healthcare students’ decisions around study abroad programmes. Nurse Educ. Today. 2016; 39: 63–71. 10.1016/j.nedt.2015.12.024 27006034

[11] Burgess CA, Reimer-Kirkham S, Astle B. Motivation and international clinical placements: shifting nursing students to a global citizenship perspective. IJNES. 2014; 11 (1): 1–8. 10.1515/ijnes-2013-0056 24739330

[12] Casey D, Murphy K. Irish nursing students’ experiences of service learning. Nurs Health Sci. 2008; 10 (4): 306–311. 10.1111/j.1442-2018.2008.00409.x 19128307

[13] Chen M, Bang H. Exploring East Asian Undergraduate Students’ Perceptions regarding the Effectiveness of their Preparation for Study Abroad in U.S. Universities. J. Int. Students. 2020; 10(3). 10.32674/jis.v10i3.754.

[14] Goodman B, Jones R, Macias MS. An exploratory survey of Spanish and English nursing students’ views on studying or working abroad. Nurse Educ. Today. 2008; 28 (3): 378–384. 10.1016/j.nedt.2007.06.013 17825457

[15] Landon AC, Tarrant MA, Rubin DL, Stoner L. Beyond “Just Do It”: Fostering Higher-Order Learning Outcomes in Short-Term Study Abroad. Area Open. 2017; 31 (1): 1–7. 10.1177/2332858416686046.

[16] Reynolds-Case A. The value of short-term study abroad: an increase in students’ cultural and pragmatic competency. Foreign Lang Ann. 2013; 46(2): 311–322. 10.1111/flan.12034.

[17] Hamad R, Lee CM. An assessment of how length of study-abroad programs influences cross-cultural adaptation. J Hum Behav Soc Environ. 2013; 23(5): 661–674. 10.1080/10911359.2013.788461.

[18] Nguyen A. Intercultural Competence in Short-Term Study Abroad. Frontiers: The Interdisciplinary Journal of Study Abroad. 2017; 29(2): 109–127. https://www.frontiersjournal.com.

[19] Philips L, Bloom T, Gainey T, Chiocca E. Influence of Short-Term Study Abroad Experiences on Community Health Baccalaureate Students. J. Nurs. Educ. 2017; 56(9): 528–533. 10.3928/01484834-20170817-03 28876438

[20] Chen YC. Essential professional core competencies for nurses. Journal of Nursing. 2010; 57(5), 12–17. (Chinese). 20878605

[21] Blumer H. Symbolic Interactionism: Perspective and Method. New Jersey, Englewood Cliffs: Prentice-Hall, Inc., 1969, 1–61p.

[22] Bengtsson M. How to plan and perform a qualitative study using content analysis. NursingPlus Open. 2016; 2: 8–14. 10.1016/j.npls.2016.01.001.

[23] Lincoln YS, Guba EG. Naturalistic Inquiry. Newbury Park, CA: SAGE Publications, Inc., 1985, 289–331p.

[24] Gabowska S, Wearing S, Lyons K, Tarrant M, Landon A. A rite of passage Exploring youth transformation and global citizenry in the study abroad experience. Tour. Recreat. Res. 2017; 1–11. 10.1080/02508281.2017.1292177.

[25] Payne EK, Chapman H, Daly A, Darby S, Heft M. Short-term study abroad: the students’ perspective on London. ATEJ. 2019; 14(4):269–274. 10.4085/1404269.

[26] Nagabandi A., Clavera I., Liu S., Fearing R S., Abbeel P., Levine S., et al. Learning to adapt in dynamic, real-world enviornments through meta-reinforcement learning. Conference paper at ICLR 2019. 2018; https://arXiv:1803.11347.

[27] Mikkonen K, Pitkäjärvi M, Kääriäinen M. International Healthcare Students in Clinical Learning Environments. In: Barton G., Hartwig K. (eds) Professional Learning in the Work Place for International Students. Professional and Practice-based Learning. 2017; 19. 10.1007/978-3-319-60058-1_9.

[28] Lee HJ, Lee J, Makar KA, Fishman BJ, Teasley SD. A cross-cultural comparison of college students’ learning strategies for academic achievement between South Korea and the USA. Stud High Educ. 2017; 42 (1): 169–183. 10.1080/03075079.2015.1045473.

[29] Wong LLC, Nunan D. The learning styles and strategies of effective language learners. System. 2011; 39: 144–163. 10.1016/j.system.2011.05.004.

[30] Bonazzo C, Wong YJ. Japanese international female students’ experience of discrimination, prejudice, and stereotypes. Coll Stud J. 2007; 41 (3): 631–639.

[31] Wang CC, Andre K, Greenwood KM. Chinese students studying at Australian universities with specific reference to nursing students: A narrative literature review. Nurse Educ. Today. 2015; 35: 609–619. 10.1016/j.nedt.2014.12.005 25537169

[32] Trower H, Lehmann W. Strategic escapes: Negotiating motivations of personal growth and instrumental benefits in the decision to study abroad. Brit. Educ Res J. 2017; 43 (2): 275–289. 10.1002/berj.3258.

[33] Laslo-Roth R. The Power of Change and the Need to Change Power: Changing Perception of Power in the Organizational Setting. In: Muhlbauer V., Harry W. (eds) Redefining Management. 2017; Springer, Cham. 10.1007/978-3-319-69209-8_2.

[34] Wojciuk A. Handbook of cultural security. Chp 18: Higher education as a soft power in international relations. 2018; 343–360. 10.4337/9781786437747.00025.

[35] Reid A. Internationalisation, Inclusivity and Learning. In C. M. Wong, K. P. Mohanan, and D. Pan (Eds.), Proceedings of the Second Symposium on Teaching and Learning in Higher Education. 2002. (pp. 327–332). National University of Singapore.

[36] Briney A. Geography of Australia-Learn Geographic Information about Australia. 2017; https://www.thoughtco.com/geography-of-australia-1434351 (accessed 25 May2020).

[37] Chang L, Miranda CKM, Li T, Wu BP, Chen BB, Lu HJ. Cultural Adaptations to Environmental Variability: An Evolutionary Account of East–West Differences. Educ Psychol Rev. 2011; 23 (1): 99–129. 10.1007/s10648-010-9149-0.

