# Supplementary material for: An enjoyable involvement: A qualitative study of short-term study abroad for nursing students
Source: PLoS One. 2021 Apr 2;16(4):e0249629. doi: 10.1371/journal.pone.0249629 (PMC8018622; doi:10.1371/journal.pone.0249629)
Supplement: S1 Table — (DOCX) [file pone.0249629.s001.docx]

Table 1

*An enjoyable involvement of studying abroad*

| The self in relation to the world |
| --- |
| 1. Relating to the world 2. Adapting to a new environment 3. Gaining a deeper understanding of oneself 4. Acquiring a new vision |
| Learning diversity |
| 1. Motivating oneself to change 2. Inspiring self-confidence 3. Reenergizing nursing education |
| A once-in-a-lifetime experience |
| 1. Facing the reality 2. Confidence, personal growth, and knowing before doing 3. Learning from the real world |
