# Supplementary material for: An enjoyable involvement: A qualitative study of short-term study abroad for nursing students
Source: PLoS One. 2021 Apr 2;16(4):e0249629. doi: 10.1371/journal.pone.0249629 (PMC8018622; doi:10.1371/journal.pone.0249629)
Supplement: S2 Table — (DOCX) [file pone.0249629.s002.docx]

Table 2

*Participants demographics information*

|  | Age | Academic | No. of interview |
| --- | --- | --- | --- |
| S1 | 23 | BSN | IBO & IAO |
| S2 | 23 | BSN | IBO & IAO |
| S3 | 22 | BSN | IBO & IAO |
| S4 | 37 | Part-time in Master | IBO & IAO |
| S5 | 29 | Part-time in Master | IBO & IAO |
| S6 | 21 | BSN | IBO & IAO |
| S7 | 22 | BSN | IAO |
| S8 | 22 | BSN | IBO & IAO |
| S9 | 23 | BSN | IBO & IAO |
| S10 | 22 | BSN | IBO & IAO |
| S11 | 23 | BSN | IAO |
| S12 | 23 | BSN | IAO |
| S13 | 22 | BSN | IAO |
| S14 | 23 | BSN | IAO |

IBO: Interview before oversea; IAO: Interview after oversea
