# Supplementary material for: An enjoyable involvement: A qualitative study of short-term study abroad for nursing students
Source: PLoS One. 2021 Apr 2;16(4):e0249629. doi: 10.1371/journal.pone.0249629 (PMC8018622; doi:10.1371/journal.pone.0249629)
Supplement: S1 File — (DOCX) [file pone.0249629.s003.docx]

**Interview Guide**

1. Please tell me a little about yourself?
2. Could you please share your experience of overseas study?

2. If you are going to describe your study abroad experience to a friend or family member, what would you say?

3. What have been the most important things about this experience that you want to share with your friends and family?

4. What experience had the most impact on you personally, and why?

5. What was the most significant thing you learned about yourself through your study abroad experience? Why?

6. What are the lessons you learned that you never want to forget?

7. How do you think your study abroad experience will aid you in the future?

8. What was your social interaction like with others when you were abroad?

9. Who had the most significant impact on you while you were abroad? Why?

10. Share a challenging experience where you had to resolve a conflict or solve a problem. What did you learn about yourself through that experience?

11. Share an example of a time when you may have been in danger or afraid. What did you learn from it? Why?

12. How did learning about and living in a different culture change your perception of your host culture?

**訪談指引 (學習前 before trip)**

1. 可不可以自我介紹一下?
2. 可否分享一下你遴選海外學習歷程?
3. 如果你將告訴你朋友或家人，你即將進行海外學習，你會說什麼?
4. 你預期你會與你朋友或家人分享什麼樣的學習經驗?
5. 你想海外學習對你個人會有何影響? 為甚麼?
6. 你覺得海外學習經驗對你的專業(護理)學習有何幫助或影響?為甚麼?
7. 你預期哪一些的學習會最令你難忘?
8. 你覺得你的海外學習經驗對你將來專業發展(護理)影響為何?
9. 在國外時，你預期誰對你的社交人際關係會有影響? 為甚麼?
10. 在國外時，你預期誰對你生活或學習影響最深? 為甚麼?
11. 分享一項，你在海外可能會感覺到挑戰最大，或衝突最大的事情?你覺得你會如何面對與解決?你預期從這衝突中你會學到什麼?
12. 在海外期間，你預期你可能會感到最害怕或危險的事情?你預期你從中學習可以學到什麼？為甚麼?
13. 不同的文化，不同的學習型態，你做了甚麼準備來適應你原有的學習型態(習慣)及生活認知?

**訪談指引(學習後 after trip)**

1. 可不可以自我介紹一下? (可省略)
2. 可否分享一下你去海外的學習經驗?
3. 你現在即將告訴你朋友或家人你的海外學習經驗，你會說什麼?
4. 什麼樣的學習經驗你一定要與你的朋友或家人分享?
5. 此趟海外學習對你個人的影響為何? 為甚麼?
6. 你覺得，海外學習經驗對你本身的專業學習(護理)有何幫助?為甚麼?
7. 哪一些的學習是最令你難忘的?
8. 你覺得你的海外學習經驗對你的將來的影響為何? 對專業發展(護理)影響為何?
9. 在國外時，誰對你的社交人際關係影響最深? 為甚麼?
10. 在國外時，誰對你的學習或生活影響最深? 為甚麼?
11. 分享一項，你在海外時感覺挑戰最大或衝突最大的事情?你如何面對與解決?從這衝突中你學到什麼?
12. 有無/形容在海外期間，你感到最害怕或危險的事情?你從中學習到什麼？為甚麼?
13. 不同的文化，不同的學習型態有無/如何改變你原有的學習及生活認知?
